# Fluoxetine Disrupts Ovarian Serotonin Signaling and Oocyte Competence in Mice

**DOI:** 10.3390/ph18111647

**Published:** 2025-10-31

**Authors:** Nina M. Alyoshina, Maria V. Beketova, Maria D. Tkachenko, Yulia O. Nikishina, Veronika S. Frolova, Lyudmila A. Malchenko, Maria L. Semenova, Maria P. Rubtsova, Denis A. Nikishin

**Affiliations:** 1 Koltzov Institute of Developmental Biology, 26 Vavilov Street, Moscow 119334, Russia; ninalyoshina@gmail.com (N.M.A.); grachsmeliy@gmail.com (M.V.B.); tkmadm@yandex.ru (M.D.T.); zubova.y@gmail.com (Y.O.N.); ludmilamalchenko@gmail.com (L.A.M.); 2 Department of Embryology, Biological Faculty, Lomonosov Moscow State University, Lenin Hills, 1/12, Moscow 119234, Russia; frolova.veronika.2014@post.bio.msu.ru (V.S.F.); mlsemenova@gmail.com (M.L.S.); 3 Department of Chemistry of Natural Compounds, Faculty of Chemistry, Lomonosov Moscow State University, Lenin Hills, 1/3, Moscow 119234, Russia; mprubtsova@gmail.com

**Keywords:** fluoxetine, ovary, oocyte competence, GDF9, serotonin, SERT (Slc6a4), fertility

## Abstract

**Background:** Selective serotonin reuptake inhibitors (SSRIs) are widely prescribed, yet their direct impact on ovarian function remains poorly understood. While serotonin signaling is known to occur within the ovarian follicle, the specific molecular consequences of its disruption by SSRIs are unclear. This study aimed to elucidate the direct, intra-ovarian mechanisms by which fluoxetine, a common SSRI, affects follicular development and oocyte competence. **Methods:** We administered fluoxetine (20 mg/kg) or vehicle daily for seven days to both prepubertal and adult female mice to model short-term therapeutic exposure. **Results:** Fluoxetine treatment successfully blocked peripheral serotonin uptake, reducing serum levels by over 90%. Crucially, this occurred without altering circulating levels of estradiol, FSH, or LH and without disrupting the estrous cycle, indicating a mechanism independent of the central hypothalamic–pituitary–gonadal axis. Instead, we pinpoint a direct ovarian effect: fluoxetine inhibited serotonin transport activity in oocytes and significantly downregulated the expression of the pivotal oocyte-derived growth factor *Gdf9*. This was accompanied by reduced expression of genes crucial for granulosa cell function (*Lhr*, *Fshr*) and steroidogenesis (*Cyp19a1*). Functionally, these molecular changes manifested as a decline in oocyte quality and a significant reduction in ovulation rates in adult mice. Notably, these detrimental effects were more pronounced in prepubertal mice, indicating a heightened vulnerability during early follicular development. **Conclusions:** Our findings reveal a direct, intra-ovarian mechanism of fluoxetine-induced disruption. By inhibiting oocyte serotonin transport and downregulating GDF9, fluoxetine impairs critical oocyte–granulosa cell communication, thereby compromising oocyte competence and reducing fertility outcomes. This work identifies follicular development as a critical window of susceptibility to SSRI exposure, holding significant clinical implications for reproductive-aged and adolescent populations.

## 1. Introduction

The female reproductive system is highly susceptible to external factors, which can impact not only maternal health but also the development and well-being of future offspring. In this context, the effects of widely prescribed psychotropic drugs are of particular concern. Women are twice as likely as men to experience depression, with a high incidence during their reproductive years [1]. Consequently, selective serotonin reuptake inhibitors (SSRIs) are commonly prescribed. However, their safety for the female reproductive system remains a subject of ongoing debate [2,3,4,5,6,7,8], necessitating further study to understand their short- and long-term effects.

The primary antidepressant mechanism of SSRIs involves enhancing serotonergic neurotransmission by increasing serotonin (5-hydroxytryptamine, 5-HT) levels in the synaptic cleft [9]. Beyond the central nervous system (CNS), however, serotonin has a much broader physiological role. Notably, only 5% of the body’s serotonin is synthesized in the brain, with the remaining 95% produced in peripheral tissues [10,11]. A growing body of evidence indicates that serotonin is also a key signaling molecule in female reproduction, with its presence detected in the oviduct, uterus, ovaries, oocytes, and early mammalian embryos [12,13,14,15,16,17]. This has prompted investigations into the local ovarian serotonergic system, revealing the expression of key components: the serotonin synthesis enzymes Tph1, Tph2, and Ddc [12,17]; the degradation enzyme Mao [18]; vesicular transporters Vmat1 and Vmat2 [19,20,21]; and various classes of serotonin receptors (Htr) [12,20,21]. Intriguingly, the direct target of SSRIs, the serotonin reuptake transporter (Sert/Slc6A4), is also expressed and active in ovarian tissues, particularly in follicles [17]. Pharmacological or genetic inhibition of Sert has been shown to reduce aromatase (Cyp19a1) expression and subsequent 17β-estradiol (E2) secretion [22,23,24]. In contrast, serotonin itself appears to stimulate steroidogenesis in mammalian follicles and granulosa cell cultures [25,26,27]. However, the precise mechanisms by which SSRIs and serotonin modulate steroid hormone synthesis and overall ovarian function remain to be fully elucidated.

SSRIs are known to alter serotonin concentrations not only in the CNS but also in the blood and peripheral tissues [28,29], potentially leading to functional disturbances. Our previous work demonstrated that mouse oocytes accumulate serotonin via Sert [17] and that oocyte Sert activity correlates with follicle survival and selection [30]. Moreover, fluoxetine treatment leads to tangible reproductive consequences, including reduced oocyte competence (the oocyte’s ability to resume meiosis, fertilize, and develop into a viable embryo) and lower ovulation rates [31]. This Sert-mediated serotonin uptake is prominent in growing follicles, a key process during both prepubertal development and adult cyclicity. Therefore, the aim of this study was to investigate the effects of short-term exposure to fluoxetine, one of the most commonly prescribed SSRIs, on ovarian function. We utilized two critical developmental windows: the first wave of folliculogenesis in prepubertal mice (7–14 days postpartum, dpp) and the established cyclicity in sexually mature adult females (2 months old).

## 2. Results

### 2.1. Short-Term Fluoxetine Administration Drastically Reduces Serum Serotonin Without Affecting Estradiol Levels

**Figure 1:**
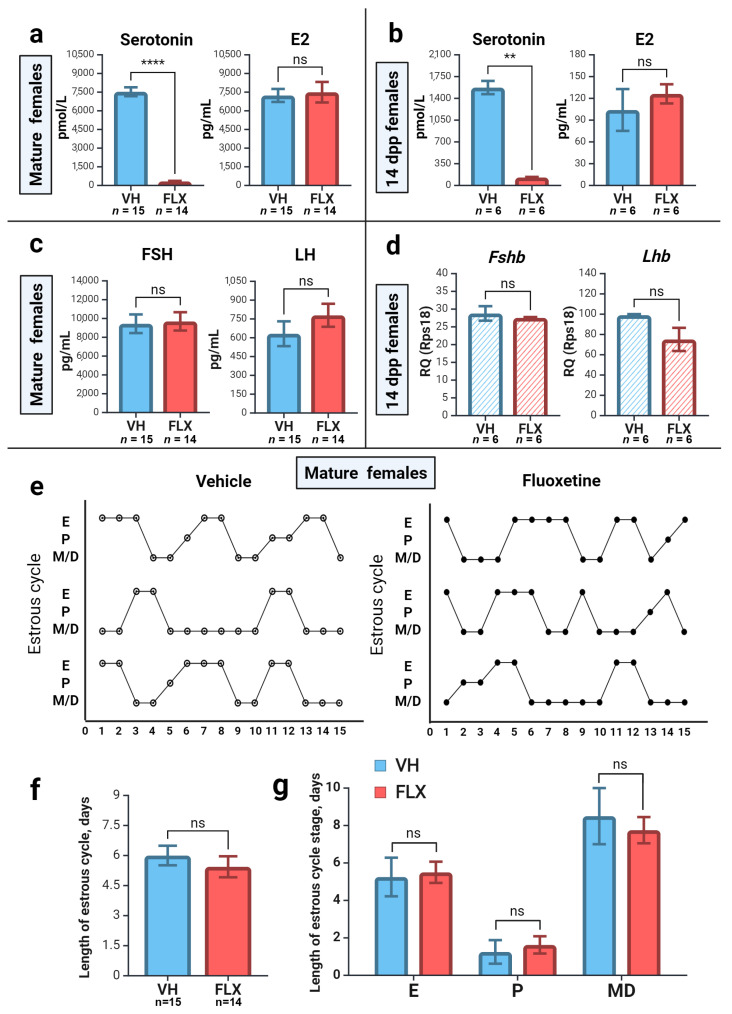
Establishment of the experimental model on mice and effects of 7 days of fluoxetine treatment on hormones. VH—vehicle treated, FLX—fluoxetine treated. (**a**,**b**) Serotonin and E2 levels in the blood serum of mature (**a**) and prepubertal (**b**) females in the experiment (M ± SEM). Significance levels are indicated as **—*p* < 0.01, ****—*p* < 0.0001 according to the Mann–Whitney U test. (**c**) Gonadotropin protein assessment in the blood serum of mature mice (M ± SEM). ns—not significant according to the Mann–Whitney U test. (**d**) Gonadotropins β-subunit gene expression in the pituitary gland of prepubertal mice. The relative quantity (RQ) was calculated using the 2^−∆Ct^ method relative to the reference gene *Rps18* (M ± SEM). ns—not significant according to the Mann–Whitney U test. (**e**) Estrous cycle of control (vehicle, *n* = 4) and fluoxetine treated (*n* = 8) mice. (**f**) Statistical evaluation of the length of the estrous cycle in mature mice (M ± SEM). ns—not significant according to the Mann–Whitney U test. (**g**) Comparison of the percentage of time spent by mice in each estrous cycle stage during 15 days of the experiment (M ± SEM). ns—not significant according to the Mann–Whitney U test. Figure created with BioRender.com (accessed on 10 September 2025).

To establish the systemic effects of fluoxetine in our experimental models, we first measured serotonin (5-HT) concentrations in the blood serum of prepubertal (14 dpp) and adult (2-month-old) female mice after a 7-day treatment course. High-performance liquid chromatography (HPLC) analysis revealed a dramatic reduction in serum 5-HT in the fluoxetine-treated groups compared to vehicle controls. Specifically, 5-HT levels decreased by ~92% in prepubertal females and ~96% in adult females (Figure 1a,b, left panels). It was also noted that basal 5-HT levels in control prepubertal mice were approximately five-fold lower than in control adult mice.

Next, we measured serum 17β-estradiol (E2) levels by ELISA. Despite the profound drop in peripheral serotonin, the 7-day fluoxetine exposure had no significant effect on circulating E2 concentrations in either age group (Figure 1a,b, right panels). As expected, basal E2 levels were substantially lower in prepubertal mice than in adults. These results confirm that our administration protocol effectively depletes peripheral serotonin, providing a robust model to investigate the consequences of reduced systemic 5-HT on ovarian function, independent of acute changes in circulating E2.

### 2.2. Fluoxetine Administration Does Not Alter Gonadotropin Levels or Estrous Cyclicity

To determine if fluoxetine’s effects were mediated by the hypothalamic–pituitary–gonadal (HPG) axis, we assessed gonadotropin levels. In adult females, ELISA measurements showed no significant differences in the serum levels of follicle-stimulating hormone (FSH) or luteinizing hormone (LH) between fluoxetine-treated and control groups (Figure 1c). Similarly, in prepubertal mice, real-time PCR analysis of pituitary glands revealed no change in the mRNA expression of the beta-subunits of *Fsh* and *Lh* (Figure 1d).

Consistent with the stable gonadotropin levels, the estrous cycle in adult females was unaffected by fluoxetine treatment. We observed no significant differences in the overall length of the estrous cycle (Figure 1f) or the duration of its individual stages (Figure 1e,g). Together, these data strongly suggest that the effects of short-term fluoxetine exposure are not mediated by central gonadotropic regulation but likely stem from a direct action on the ovary.

### 2.3. Fluoxetine Disrupts Serotonin Accumulation in Oocytes of Growing Follicles Without Altering Follicular Morphology

**Figure 2:**
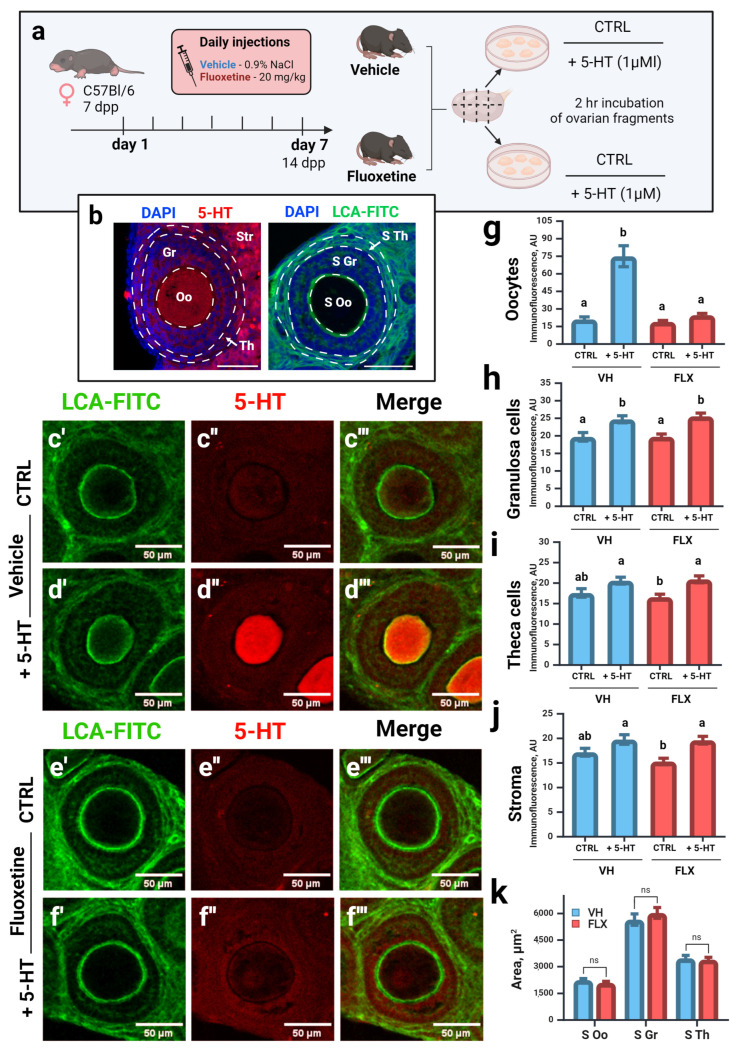
Effects of fluoxetine on serotonin accumulation in the ovary and follicular morphology. VH—vehicle treated, FLX—fluoxetine treated. (**a**) Experiment design. CTRL—control, incubation without extra chemicals, +5-HT—incubation with 1 μM of serotonin. *n* = 48 ovarian fragments for each group. (**b**) Schematic representation of the zones of interest. CLSM microphotographs of ovarian tissue (growing preantral follicles) labeled with antibodies against serotonin (5-HT) and with Lens Culinaris agglutinin conjugated with FITC (LCA-FITC), nuclei were marked with DAPI. Oo—oocyte, Gr—granulosa cells, Th—theca cells, Str—stromal tissue, S Oo—area of oocyte, S Gr—area of granulosa cell layer, S Th—area of theca cell layer. Scale bar 50 μm. (**c′**–**f″′**) Morphology and serotonin accumulation in ovarian tissue of experimental animals after incubation in the presence of 1μM of 5-HT. (**g**–**j**) Quantification of anti-serotonin immunoreactivity in different compartments of the ovarian follicle (M ± SEM)—in oocytes (**g**), granulosa cells (**h**), theca cells (**i**) and stroma (**j**). AU—arbitrary units of immunofluorescence. Letters a and b indicate significant differences (*p* < 0.05) between groups using the Kruskal–Wallis test with Dunn’s multiple comparisons test. (**k**) Quantification of the area of growing follicle compartments (M ± SEM). ns—not significant according to the Mann–Whitney U test. Figure created with BioRender.com (accessed on 10 September 2025).

Given the evidence for a direct ovarian effect, we assessed the capacity for serotonin accumulation in ovarian compartments using an ex vivo culture system with ovaries from 14 dpp females, which are enriched with growing preantral follicles (Figure 2a). Ovarian fragments were incubated with 5-HT, and its uptake was visualized via immunostaining (Figure 2c–f).

As expected, oocytes from control animals exhibited robust 5-HT accumulation (Figure 2d″,g). In contrast, this accumulation was nearly abolished in oocytes from fluoxetine-treated females (Figure 2f″,g), consistent with both the systemic serotonin depletion and direct inhibition of the oocyte’s serotonin transporter (Sert). Other ovarian compartments, such as granulosa and theca cell layers, showed minimal 5-HT accumulation with no significant differences between groups (Figure 2h–j), suggesting the involvement of transport mechanisms insensitive to fluoxetine in these cells.

We then assessed follicular morphology in these preantral follicles (80–130 μm diameter). Analysis of the cross-sectional area of the oocyte, granulosa layer, and theca layer revealed no significant differences between the control and fluoxetine-treated groups (Figure 2b,k). This indicates that at this time point, fluoxetine impairs a key oocyte function—serotonin uptake—rather than inducing gross morphological changes in the follicle.

### 2.4. Fluoxetine Alters the Expression of Key Ovarian Genes

To dissect the molecular mechanisms underlying fluoxetine’s impact, we performed qPCR analysis on a curated set of genes that reflect the functional status of oocytes and somatic follicular cells.

#### 2.4.1. Fluoxetine Downregulates the Expression of Key Oocyte-Secreted Factors and Sert

**Figure 3:**
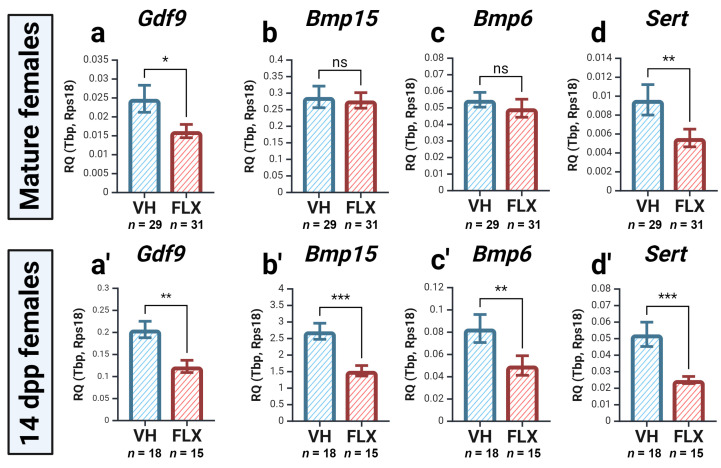
Expression of genes encoding oocyte factors after 7 days of fluoxetine treatment in the ovaries of mature (**a**–**d**) and prepubertal (**a′**–**d′**) females. VH—vehicle treated; FLX—fluoxetine treated. The relative quantity (RQ) was calculated using the 2^−ΔCt^ method relative to the reference genes *Tbp* and *Rps18* (M ± SEM). Significance levels are indicated as *—*p* < 0.05, **—*p* < 0.01, ***—*p* < 0.001, ns—not significant according to the Mann–Whitney U test. Figure created with BioRender.com (accessed on 10 September 2025).

We first evaluated the expression of critical oocyte-derived factors that orchestrate folliculogenesis. Expression of *Gdf9* was significantly downregulated in both adult and prepubertal mice following fluoxetine treatment, with a notable and consistent effect observed in both groups (Figure 3a,a′). Similarly, the expression of *Bmp15* and *Bmp6* was significantly reduced in prepubertal animals, although no significant change was observed in adults (Figure 3b,c,b′,c′).

We next examined the expression of the fluoxetine target gene itself, *Sert* (*Slc6a4*). Interestingly, *Sert* mRNA levels were significantly decreased in the ovaries of both adult and prepubertal mice after fluoxetine treatment (Figure 3d,d′). This suggests the existence of a positive feedback mechanism where Sert activity may regulate its own transcription.

#### 2.4.2. Fluoxetine Alters the Expression of Genes for Steroidogenesis and Gonadotropin Response

**Figure 4:**
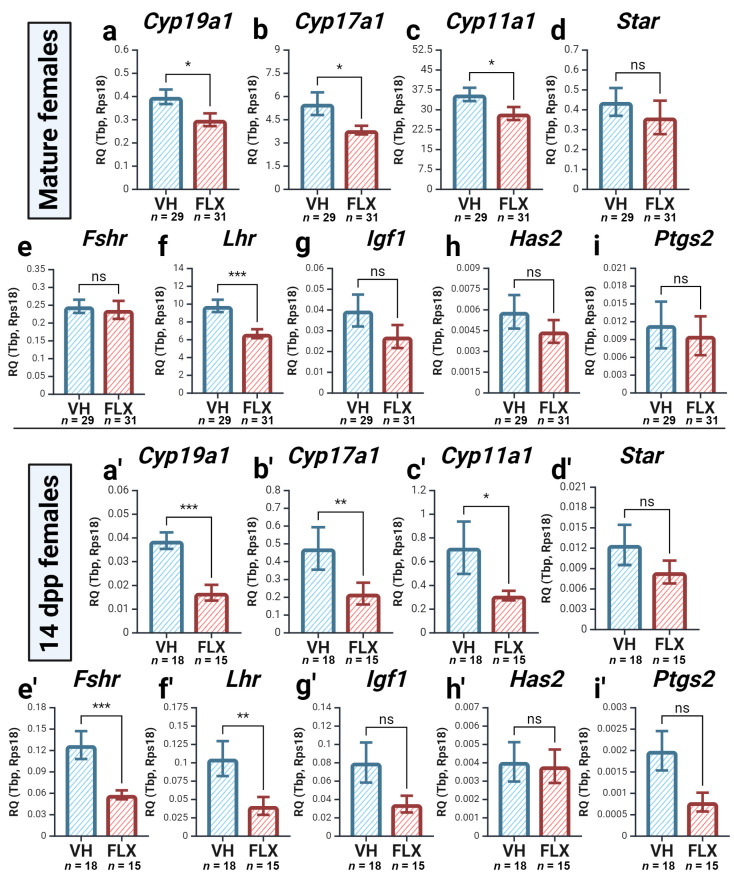
Expression of genes encoding functional state of somatic cells and steroidogenic enzymes after 7 days of fluoxetine treatment in the ovaries of mature (**a**–**i**) and prepubertal (**a′**–**i′**) females. VH—vehicle treated, FLX—fluoxetine treated. The relative quantity (RQ) was calculated using the 2^−ΔCt^ method relative to the reference genes *Tbp* and *Rps18* (M ± SEM). Significance levels are indicated as *—*p* < 0.05, **—*p* < 0.01, and ***—*p* < 0.001, ns—not significant according to the Mann–Whitney U test. Figure created with BioRender.com (accessed on 10 September 2025).

We then analyzed the expression of genes related to somatic cell function. The expression of genes crucial for both thecal androgen production (*Cyp17a1*) and granulosa cell estrogen synthesis (*Cyp19a1*) was significantly decreased in both age groups (Figure 4a,b,a′,b′). Likewise, the expression of *Cyp11a1* and *Star*, involved in the initial steps of steroidogenesis, was also significantly downregulated (Figure 4c,d,c′,d′). This downregulation of steroidogenic gene expression is particularly noteworthy given the lack of change in systemic E2 levels, suggesting the presence of compensatory mechanisms that maintain hormonal homeostasis over this short-term exposure.

Regarding gonadotropin sensitivity, the expression of *Fshr* was significantly reduced only in prepubertal mice, whereas *Lhr* expression was downregulated in both age groups (Figure 4e,f,e′,f′). Expression of *Igf1* showed a non-significant decreasing trend in both groups (Figure 4g,g′). Finally, the expression of ovulation-associated genes, *Has2* and *Ptgs2*, was not significantly affected in either model (Figure 4h,i,h′,i′). In summary, fluoxetine negatively impacts the transcriptional profile of genes essential for steroidogenesis and gonadotropin signaling, with effects often being more pronounced in the prepubertal ovary.

### 2.5. Fluoxetine Reduces Ovarian GDF9 Protein Levels

**Figure 5:**
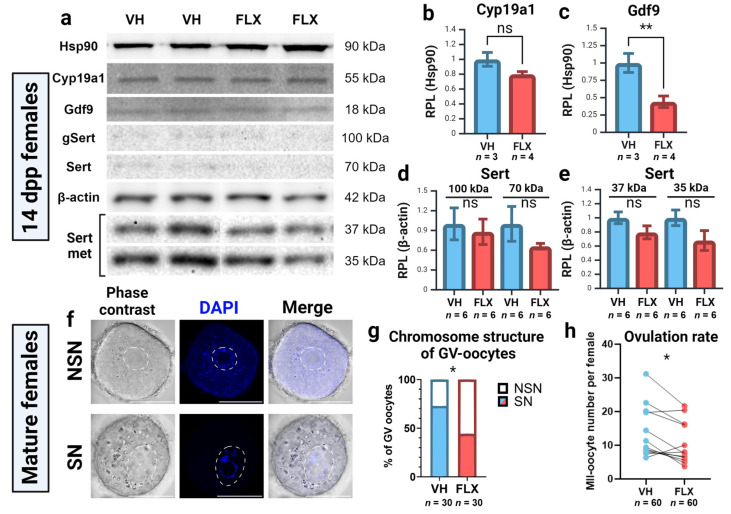
Effects of 7-day fluoxetine treatment on protein expression and parameters of oocyte quality. VH—vehicle treated, FLX—fluoxetine treated. (**a**) Western blot analysis of Gdf9, Cyp19a1 and Sert protein expression in the ovaries of 14 dpp females in the experiment. gSert—glycosylated form of Sert protein, Sert met—immunopositive metabolites of Sert. (**b**–**e**) Quantification of the results of Western blot analysis. Relative protein expression level (RPL) was calculated in relation to reference proteins, Hsp90 and β-actin (M ± SEM). *n*—the number of samples analyzed per group. Significance levels are indicated as **—*p* < 0.01, ns—not significant according to the Mann–Whitney U test. (**f**) Visualization of chromatin conformation in nucleolus-like bodies of GV-oocytes. Scale bar 50 μm. (**g**) Analysis of NSN and SN-oocyte percentage obtained from the ovaries of vehicle and fluoxetine treated mature females. (**h**) Number of ovulated MII-oocytes obtained from oviducts of experimental females (M ± SEM). Paired values denote the quantity of oocytes from one experiment. Significance level is indicated as *—*p* < 0.05 according to the Wilcoxon test. Figure created with BioRender.com (accessed on 10 September 2025).

To validate our gene expression findings at the protein level, we performed Western blot analysis on ovarian lysates from prepubertal females for GDF9, Cyp19a1 (aromatase), and Sert (Figure 5a). Consistent with the stable systemic E2 levels, Cyp19a1 protein expression was unchanged by fluoxetine treatment (Figure 5b). In contrast, and in agreement with our qPCR data, GDF9 protein levels were significantly reduced in the ovaries of fluoxetine-treated mice (Figure 5c). For Sert, while a decreasing trend was observed, no statistically significant changes were detected in the abundance of its full-length (100 kDa), non-glycosylated (71 kDa), or fragmented forms (37–35 kDa) (Figure 5d,e). These results identify the downregulation of the critical oocyte-derived factor GDF9 as a key molecular consequence of fluoxetine exposure in the prepubertal ovary.

### 2.6. Fluoxetine Impairs Oocyte Competence and Reduces Ovulation Rate

To determine if the observed molecular changes translated to functional deficits, we assessed oocyte quality and ovulatory outcomes in adult mice. First, we evaluated germinal vesicle (GV)-stage oocytes retrieved after PMSG stimulation. Fluoxetine treatment led to a significant decrease in the proportion of developmentally competent oocytes with surrounded nucleolus (SN-type) and a corresponding increase in lower-quality non-surrounded nucleolus (NSN-type) oocytes (Figure 5f,g).

Furthermore, following a full superovulation protocol, fluoxetine-treated females yielded a significantly lower number of metaphase II (MII) oocytes compared to controls (Figure 5h). Collectively, these data demonstrate that the molecular and functional alterations induced by fluoxetine translate into a tangible decline in both oocyte quality and ovulatory potential.

### 2.7. Fluoxetine-Induced Oocyte Damage Is Independent of Oxidative Stress and Telomere Maintenance Pathways

**Figure 6:**
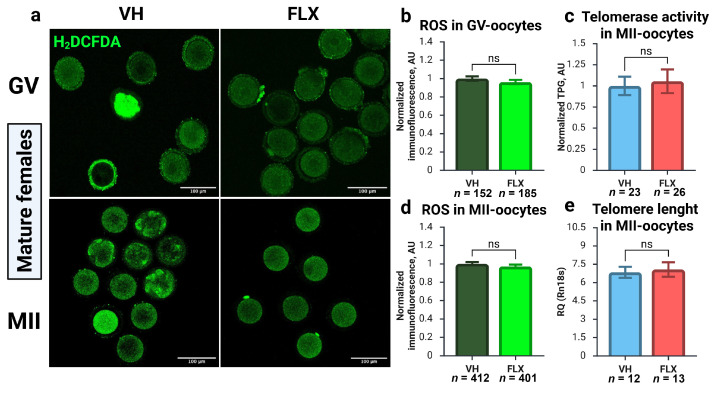
Effects of 7-day fluoxetine treatment on ROS production and telomere maintenance machinery in GV- and MII-oocytes. VH—vehicle treated, FLX—fluoxetine treated. (**a**) CLSM microphotographs of GV- and MII-oocytes showing detection of intracellular ROS with a fluorescent probe 6-carboxy-H_2_DCFDA. (**b**,**d**) Quantification of ROS immunoreactivity in GV- and MII-oocytes obtained from experimental animals (M ± SEM). AU—arbitrary units of immunofluorescence, values are normalized to the mean value in the VH group. (**c**) Telomerase activity in MII-oocytes from experimental mice, analyzed by TRAP-qPCR and normalized to the values of the VH group. TPG—telomerase product generated, AU—arbitrary units. (**e**) Relative telomere length in MII-oocytes, calculated using the 2^−ΔCt^ method relative to the reference gene *Rn18s* (M ± SEM). ns—not significant according to the Mann–Whitney U test. Figure created with BioRender.com (accessed on 10 September 2025).

To elucidate the mechanisms underlying the decline in oocyte quality, we investigated two pathways known to impact gamete viability: oxidative stress and telomere maintenance (Figure 6). Quantification of reactive oxygen species (ROS) via H_2_O_2_ levels in GV-stage oocytes revealed no significant difference between fluoxetine-treated and control groups (Figure 6a,b). This was also true for MII oocytes matured in vivo (Figure 6a,d), indicating that fluoxetine does not induce a state of oxidative stress in the oocyte.

Next, we assessed telomere integrity. Telomerase activity in MII oocytes was not significantly different between the treatment and control groups (Figure 6c). Moreover, qPCR analysis of telomere length also showed no evidence of fluoxetine-induced telomere shortening in MII oocytes (Figure 6e). Taken together, these results strongly suggest that the detrimental impact of fluoxetine on oocyte competence is not mediated by the induction of ROS-related oxidative stress or the disruption of telomere maintenance pathways.

## 3. Discussion

Research into the effects of various factors on ovarian function is essential to modern biology, medicine, and pharmacology. The ovary is not merely a component of the endocrine system; it houses the oocytes, which contain the molecular foundation for the development of a future organism and are themselves susceptible to external influences. Our recent work revealed that chronic fluoxetine exposure functionally impairs reproduction by reducing oocyte competence and ovulation rates [31]. However, that study raised a pivotal question: what are the direct, intra-ovarian molecular mechanisms driving this dysfunction? The present study serves as a direct mechanistic follow-up designed to answer this question. Our study, employing two distinct mouse models representing prepubertal and mature stages of sexual development, provides a comprehensive investigation into the effects of the widely used antidepressant fluoxetine on ovarian health, folliculogenesis, and oocyte quality.

The validity of any pharmacological model hinges on the precise calibration of its variables. We designed our experiments to specifically probe the effects of fluoxetine administration on folliculogenesis during distinct physiological periods. In the prepubertal model, the treatment window (7–14 dpp) was chosen to coincide with the first wave of follicular growth, initiated around 5–7 dpp [32,33]. This allowed us to isolate fluoxetine’s impact on the early, gonadotropin-independent stages of folliculogenesis, prior to antrum formation, active steroidogenesis, and ovulation. Conversely, the adult model targeted follicles recruited from the mature primordial follicle pool, which differ from those of the first wave [34]. In these animals, an 8-day exposure ensured that at least one full estrous cycle was affected and that both early-stage and preovulatory follicles were exposed [35]. Despite the fact that exposure was short-term, we already saw prominent effects on gene expression. Utilizing a short-term course of treatment also allowed us to isolate initial direct ovarian effects from potential long-term adaptive or central nervous system-mediated changes. To standardize the analysis, the cycle of adult mice was synchronized with PMSG prior to tissue collection.

The selected fluoxetine dosage of 20 mg/kg is another critical feature of our model, as it achieves plasma concentrations in mice that are comparable to the therapeutic levels observed in patients undergoing Prozac therapy (600–700 ng/mL), including adolescents [36,37,38]. Although the dose of 20 mg/kg/day suggested by Dulawa [36] can provoke high concentrations of fluoxetine and its metabolites in plasma, it can still be used in animal models, as the effective dose for anxiety and depression modeling, especially in juvenile mice [39]. The inclusion of a prepubertal model in our work is particularly relevant given the increasing use of SSRIs in pediatric and adolescent therapy [40], as well as the risks associated with uncontrolled consumption.

A primary systemic effect observed in both models was a dramatic decrease in serum serotonin levels. This finding is consistent with studies of non-acute SSRI exposure in humans, which report similar reductions in plasma and platelets [28,29,41,42]. We hypothesize this occurs due to systemic Sert inhibition, including in gut enterocytes and platelets, leading to rapid hepatic metabolism of free serotonin. This indicates that our model captures not only the direct effects of fluoxetine but also the physiological consequences of peripheral serotonin depletion. Notably, and in contrast to some previous research, we observed no significant effect on circulating estradiol (E2) levels. To further dissect the mechanism, we analyzed FSH and LH levels and found them to be unchanged, a finding corroborated by the lack of alterations in estrous cyclicity. Even in prepubertal mice, where we anticipated potentially subtler effects and measured pituitary *Fshb* and *Lhb* expression, no changes were detected. Collectively, these data strongly suggest that the observed ovarian detriments are not mediated by the central hypothalamic–pituitary–gonadal axis but rather stem from direct effects within the ovary.

To assess whether these direct effects manifested as morphological changes, we analyzed growing follicles in prepubertal mice. The 14 dpp timepoint is ideal for such analysis due to the homogeneity of the follicular population, which predominantly consists of primordial and early growing follicles at a uniform, FSH-independent stage (80–130 μm diameter) [43]. This allows for standardized quantification of oocyte, granulosa, and theca volumes. However, our analysis revealed no differences in these morphological parameters between experimental groups. This lack of gross structural change suggested that the effects of fluoxetine and low serotonin are more subtle, impacting follicles at a molecular and functional level.

We have previously demonstrated the presence of active serotonin transport in ovarian tissue, primarily within growing oocytes [17] and to a lesser extent in somatic follicular cells [18]. In the current study, we confirmed that in vivo fluoxetine treatment significantly ablates serotonin (5-HT) accumulation in the oocytes of growing follicles. This finding is of significant concern because these first-wave follicles are developmentally competent and contribute to the ovulatory pool [33]. Furthermore, serotonin-mediated signaling is a phylogenetically conserved mechanism involved in oocyte maturation across diverse species, including mammals [13,25,26]. As we recently showed that serotonin accumulation capacity increases with oocyte maturation [30], our current results confirm that SSRIs directly disrupt a fundamental physiological process within the oocyte itself.

Interestingly, fluoxetine treatment did not significantly affect Sert activity within the granulosa cell layer. While our previous short-term in vitro experiments did detect Sert and MAO activity in primary granulosa cells [18], it is possible that during the longer in vivo exposure of our current model, any effects on somatic cells are mitigated by compensatory mechanisms or non-specific transport. The consistent and robust observation of specific Sert activity in oocytes across multiple experiments validates the concept that high-affinity serotonin transport is a specialized feature of the female gamete.

Even more striking than the inhibition of Sert activity was the downregulation of its own gene expression. We also observed a trend towards decreased protein expression for both major isoforms of Sert (100 and 70 kDa) and its metabolites [43], indicating a reduction in its synthesis or an increase in its degradation. This aligns with clinical findings of reduced Sert binding sites on platelets during fluoxetine therapy [29] and cellular studies showing that Sert can be internalized and metabolized [43]. Given that Sert phosphorylation and inhibition by fluoxetine are dependent on serotonin as a substrate [44], our results may represent an unusual example of a disrupted positive feedback loop, where the transporter’s own activity is required for its sustained expression, a process unique to the oocyte’s physiology.

To identify the molecular consequences of Sert disruption, we analyzed the expression of key oocyte-derived factors: *Gdf9*, *Bmp15*, and *Bmp6*. GDF9 and BMP15 are critical for follicle maturation [45,46], with their expression increasing as follicles are recruited from the dormant pool [47]. GDF9, in particular, is essential for nearly all stages of follicular growth and ovulation [48,49], while BMP6 is another key oocyte-derived factor [50] also found in granulosa cells [51] and follicular fluid [52]. We observed a significant decrease in the expression of all three factors in prepubertal females. The more pronounced effect in the 14 dpp ovary is likely due to the higher oocyte-to-stroma ratio, which amplifies the oocyte’s transcriptional signal in whole-ovary lysates. Critically, *Gdf9* expression declined in both age groups, a result we confirmed at the protein level, strongly suggesting a direct impact of fluoxetine and/or low serotonin on this pathway. This perfectly corroborates our previous in vitro work, where serotonin supplementation increased *Gdf9* expression—an effect that was abolished by fluoxetine [19]. Given the dominant role of GDF9 in folliculogenesis in poly-ovulatory species like mice [45], we propose that this disruption of the GDF9 signaling axis is a central mechanism underlying fluoxetine-induced ovarian dysfunction.

The central finding of GDF9 downregulation raises the question of its particular mechanism: whether it is a direct consequence of SERT inhibition within the oocyte or a secondary effect of systemic serotonin depletion. While our data cannot definitively separate these two interconnected events, several lines of evidence point to a critical role for intra-oocyte serotonin signaling. Serotonin itself can act as a signaling molecule, and its transport can influence cellular energy status and gene expression. A potential upstream regulator of Gdf9 that could be affected by disrupted serotonin signaling is NOBOX, which is vital for oocyte-specific gene expression of many genes, let alone GDF9 [53]. However, there is a lack of scientific data on SRRIs’ action on NOBOX and many other regulators of ovarian function. Future studies using oocyte-specific Sert knockout models or research on direct serotonin effects on NOBOX and other factors in ovaries and oocytes will be necessary to dissect this precise mechanism of fluoxetine action.

Initially, we hypothesized that fluoxetine would primarily impact steroidogenesis. However, we found no significant change in systemic E2 levels. Despite this, we observed a significant decline in the gene expression of key steroidogenic enzymes, including *Cyp19a1*, *Cyp17a1*, and *Cyp11a1*. We attribute the disconnect between gene expression and circulating hormone levels to two factors. First, the short duration of the experiment may have been insufficient to deplete existing enzyme reserves and impact systemic E2. Second, extra-ovarian sources such as the brain, bone, and adipose tissue can contribute to and compensate for total blood E2 levels [54]. We also acknowledge that directly measuring E2 secretion from ovarian explants or primary granulosa cell cultures would have provided more definitive evidence for a local intra-ovarian steroidogenic defect. The absence of such direct functional data is a limitation of our study, and this will be an important focus of our future in vitro investigations. The observed downregulation of genes responsible for androgen synthesis is also noteworthy, as SSRIs have been reported to reduce testosterone levels [55]. While beyond the scope of this study, a drop in ovarian androgen precursors could be a contributing factor to altered steroidogenesis.

Another crucial aspect of granulosa cell function is their sensitivity to gonadotropins. We observed a prominent decrease in the expression of gonadotropin receptors. Interestingly, *Fshr* expression was reduced in prepubertal mice but not in adults. This highlights the 7–14 dpp period as a potential window of vulnerability. Follicles acquire FSH susceptibility at early stages, even before they become fully dependent on it for antral development [56,57]. Fluoxetine exposure during this critical period of the first folliculogenesis wave may disrupt the establishment of the FSH receptor system. In contrast, the stable *Fshr* expression in adult mice may reflect a more established and resilient system. In terms of *Lhr*, expression decreased in both models. As both FSHR and LHR are critical for follicle selection, steroidogenesis, and the switch to LH-dependence pre-ovulation [57,58], these results indicate that fluoxetine can compromise follicular quality by impairing gonadotropin sensitivity, particularly in the still-developing prepubertal ovary.

The culmination of normal follicular development is ovulation, a process requiring functional changes in the cumulus-oocyte complex [59]. The expression of *Has2*, which produces the hyaluronic acid backbone of the expanded matrix [60], and *Ptgs2* (Cox2), essential for LH response [61], are both induced by GDF9 signaling [62]. Despite our previous data showing serotonin stimulates these genes in vitro, we saw no changes in our current in vivo models. This is likely explained by the experimental design: prepubertal follicles lack a defined cumulus and are not yet competent for ovulation, while adult mice received only PMSG and not the ovulatory hCG trigger required to induce the expression of these periovulatory genes.

Higher vulnerability of prepubertal ovaries to fluoxetine is a critical finding. This increased sensitivity is likely multifactorial. Firstly, the higher oocyte-to-stroma ratio in prepubertal ovaries amplifies the transcriptional contribution of oocytes in whole-ovary analyses, making the downregulation of oocyte-specific genes like Gdf9 more pronounced. Secondly, the first wave of folliculogenesis represents a critical window where key signaling pathways, including the FSH receptor system, are being established. Disruption by fluoxetine during this period may have more profound and lasting effects compared to the mature, cyclically recruited follicles in adults, which operate within a more stable and resilient endocrine system. Finally, developmental differences in SERT expression, activity, or its associated regulatory networks warrant further investigation.

Ultimately, the most critical readout of any toxicant effect is the direct impact on oocyte quality and quantity. We evaluated oocytes at both the germinal vesicle (GV) and metaphase II (MII) stages. In adult mice, fluoxetine treatment caused a significant decrease in the proportion of high-quality GV-oocytes with a surrounded nucleolus (SN-type), which are known to have better developmental competence [63,64], and a corresponding increase in lower-quality NSN-type oocytes. Furthermore, we clearly demonstrated that the number of MII oocytes retrieved after superovulation was significantly reduced by fluoxetine exposure. Our analysis of ROS levels and telomere maintenance machinery suggests these negative effects are not mediated by oxidative stress or telomere disruption. Instead, these results demonstrate that even short-term fluoxetine exposure during the final stages of follicular growth has a direct, negative impact on oocyte competence and ovulatory success.

A key limitation of the current study is that we did not assess the ultimate functional endpoints of oocyte competence. While the observed decrease in SN-type oocytes and reduced ovulation rates are strong indicators of compromised fertility, a crucial next step will be to conduct in vitro fertilization studies. Assessing the fertilization capacity of fluoxetine-exposed oocytes and their subsequent potential for preimplantation embryonic development is essential to directly link the molecular disruptions we identified to tangible reproductive outcomes. It is also important to acknowledge that our study focused on a short-term exposure model to capture the initial molecular response within the ovary. This design revealed a transcriptional response in granulosa cells that was not yet reflected at the aromatase protein level or systemic estradiol levels. This suggests that the primary effect is a disruption of oocyte–granulosa cell signaling rather than an immediate impairment of steroidogenesis. This model, however, does not inform on the long-term consequences of chronic SSRI use or the potential for recovery after drug cessation. Future studies involving chronic administration and withdrawal periods are needed to fully understand the clinical implications of these findings. Finally, while our data point towards a SERT-mediated mechanism within the oocyte, it is critical to acknowledge that fluoxetine possesses known non-serotonergic activities, including interactions with sigma-1 receptors [65], which are also expressed in ovarian tissue. Comparative experiments in a Sert genetic knockout mouse model would be crucial to definitively ascertain whether the observed effects on oocyte competence are exclusively mediated by SERT inhibition or involve these alternative pathways.

While our study identifies an intraovarian mechanism of fluoxetine action in mice, it is important to consider the limitations when extrapolating these findings to human fertility outcomes. Mice are polyovulatory with different follicular dynamics and endocrine profiles compared to humans. Furthermore, human reproductive cycles are longer and more complex. Therefore, while our data highlight a potentially significant risk to the ovarian reserve and oocyte quality, future studies using human ovarian cortical tissue, granulosa cell cultures, or correlative clinical data from IVF cycles are essential to confirm the translational relevance of these findings for women of reproductive age.

In conclusion, our findings identify follicular development as a critical period of vulnerability to fluoxetine, with the oocyte itself acting as the primary target of a disruptive molecular cascade.

## 4. Materials and Methods

### 4.1. Animals and Ethical Approval

Female C57BL/6J mice were obtained from the Stolbovaya breeding facility (Moscow region, Russia) and housed in the vivarium of the Koltzov Institute of Developmental Biology, Russian Academy of Sciences (IDB RAS). Animals were maintained under standard controlled conditions (22–24 °C, 14L:10D photoperiod) with ad libitum access to food and water. All experimental procedures were performed in accordance with the Council of the European Communities Directive 86/609/EEC and were approved by the Commission on Bioethics of IDB RAS (project identification code: № 68, date: 23 March 2023).

### 4.2. Experimental Design, Drug Administration, and Sample Collection

**Figure 7:**
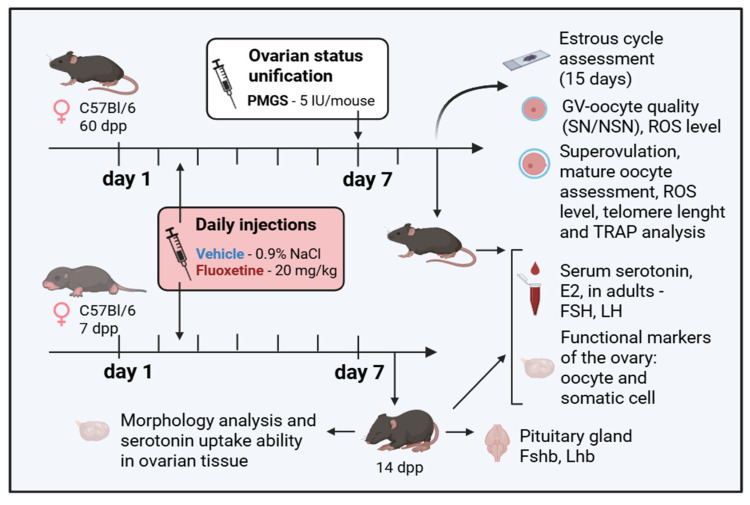
The scheme of the experiment. Mature (60 dpp) and prepubertal (7 dpp) mice were treated with fluoxetine (FLX, 20 mg/kg/day) for 8 days and for 7 days, respectively, while control groups had saline injections (vehicle, VH, 0.9% NaCl). The next day after the final fluoxetine injection, biomaterials were obtained for multiple tests. Figure created with BioRender.com (accessed on 10 September 2025).

Two age-based models were used (Figure 7): a prepubertal model, with treatment starting on postnatal day 7 (7 dpp), and a sexually mature adult model (2 months old, ~60 dpp).

Female pups starting at 7 dpp received daily subcutaneous (s.c.) injections of fluoxetine hydrochloride (FLX, F132, Sigma-Aldrich, St. Louis, MO, USA) at a dose of 20 mg/kg or an equivalent volume of saline (0.9% NaCl, vehicle) for 7 consecutive days. Biomaterials were collected on day 8 (at 15 dpp). This time point coincides with the first wave of folliculogenesis, where follicles are largely synchronized and beginning to form early antrums, eliminating the need for hormonal synchronization [32].

Mature females received daily s.c. injections of FLX (20 mg/kg) or vehicle for 8 consecutive days. To standardize the follicular stage for analysis, folliculogenesis was synchronized by a single s.c. injection of 5 IU pregnant mare serum gonadotropin (PMSG, Follimag^®^, Mosagrogen, Russia) on day 7 of treatment. Tissues and serum were collected 40 h post-PMSG injection. For the collection of ovulated metaphase II (MII) oocytes, the PMSG injection was followed 48 h later by an s.c. injection of 5 IU human chorionic gonadotropin (hCG, Chorulon^®^, Intervet International B.v., Boxmeer, Netherlands), and oocytes were collected from the oviducts 14–16 h post-hCG.

A total of 269 adult female mice and 70 prepubertal female mice were used in this study. Sample sizes were determined based on our previous work and published literature to ensure sufficient statistical power while adhering to the 3Rs principle of animal welfare. Within each age group (prepubertal and adult), animals were randomly assigned to either the control (vehicle) or experimental (fluoxetine) group. To minimize bias, experimenters performing sample collection and data analysis were blinded to the group allocations. Data points were excluded from the analysis only in cases of sample damage or processing errors.

On the day of collection, mice were humanely euthanized by cervical dislocation. Blood was collected via cardiac puncture for serum preparation, and ovaries and other tissues were harvested for subsequent analyses. The estrous cycle stage of adult mice was monitored daily by vaginal cytology as previously described [66].

### 4.3. Serum Analysis: HPLC and ELISA

Serum serotonin concentration was measured using high-performance liquid chromatography with electrochemical detection (HPLC-ED), as previously described [67]. Briefly, 20 μL of serum was deproteinized with 200 μL of 0.1 N HClO_4_ containing 3,4-dihydroxybenzylamine as an internal standard.

Serum concentrations of 17β-estradiol (E2), follicle-stimulating hormone (FSH), and luteinizing hormone (LH) were quantified using commercial enzyme-linked immunosorbent assay (ELISA) kits following the manufacturer’s protocols: ImmunoFA-Estradiol (Immunotech, Moscow, Russia), Mouse FSH ELISA Kit (ELK8859, ELK Biotechnology, Wuhan, China), and Mouse LH ELISA Kit (ELK1327, ELK Biotechnology, Wuhan, China).

### 4.4. Oocyte Collection, Staining, and Analysis

MII-oocytes within cumulus-oocyte complexes were collected from the oviductal ampullae of superovulated females. Cumulus cells were removed by brief incubation in M2 medium containing hyaluronidase. Only morphologically normal MII oocytes were used for quantification and further analysis.

GV-stage oocytes were mechanically isolated from the antral follicles of adult ovaries in M2 medium. For chromatin configuration analysis, oocytes were fixed in 4% paraformaldehyde (PFA), permeabilized with 0.1% Triton X-100 in PBS (PBST), and stained with DAPI (D9542, Merck KGaA, Darmstadt, Germany). Oocytes were classified as surrounded nucleolus (SN) or non-surrounded nucleolus (NSN) type by confocal microscopy.

For ROS assessment, live GV and MII oocytes were incubated for 30 min in medium containing 10 µM 6-carboxy-2′,7′-dichlorodihydrofluorescein diacetate (carboxy-H2DCFDA, 3290, Lumiprobe, Moscow, Russia). Oocytes were then washed and imaged immediately in glass-bottom dishes using a confocal microscope to quantify fluorescence intensity.

### 4.5. Immunohistochemistry (IHC) and Image Analysis

Ovaries were fixed overnight in 4% PFA at 4 °C, cryoprotected in a sucrose gradient (15% then 30%), embedded in Tissue-Tek O.C.T. Compound (4583, Sakura, Torrance, CA, USA), and sectioned at 10 μm. For serotonin localization, sections were blocked with 5% fetal bovine serum (FBS, Pan-Eco, Moscow, Russia) and incubated overnight at 4 °C with a primary rabbit anti-serotonin antibody (1:1000, S5545, Merck KGaA, Darmstadt, Germany). To assess in vitro serotonin uptake capacity, ovaries from 14 dpp females were fragmented and incubated for 2 h in Leibovitz’s L-15 Medium (L0230, BioSera, Nuaille, France) with 1 μM serotonin before fixation and processing as above.

Sections were subsequently incubated with a secondary antibody (CF568-conjugated anti-rabbit IgG, 1:500, SAB4600085, Merck KGaA), FITC-conjugated Lens culinaris agglutinin (LCA, 1:1000, L32475, Thermo Fisher Scientific, Waltham, MA, USA) to visualize cell boundaries, and DAPI for nuclear staining. Images were acquired using a Zeiss LSM 880 Airyscan confocal microscope with consistent settings for all samples. Image analysis was performed using FIJI software (ImageJ 2.9.0/1.54f, open source, available at https://imagej.net/software/fiji/ (URL accessed on 29 March 2025)). Serotonin immunoreactivity was quantified by measuring the mean gray value (MGV) in regions of interest (oocyte, granulosa, theca). Follicle morphometry was assessed on LCA-stained images [68].

### 4.6. Gene Expression Analysis

Total RNA was extracted from whole ovaries or pituitaries using ExtractRNA reagent (Evrogen, Moscow, Russia) and treated with DNase I (DNA-free™ Kit, AM1906, Thermo Fisher Scientific, Waltham, MA, USA). RNA was reverse transcribed using an MMLV RT kit (Evrogen). Quantitative PCR was performed on a 7500 Real-Time PCR System (Applied Biosystems, Foster City, CA, USA) using 5x qPCRmix-HS SYBR+LowRox (Evrogen). The specificity of each reaction was controlled by melting curve analysis. Relative gene expression was calculated using the 2^−ΔCt^ method, with *Rps18* and *Tbp* as reference genes. For this calculation, a uniform fluorescence threshold was applied to determine cycle threshold (Ct) values for all genes within a single experiment. The reference genes *Tbp* (TATA-box binding protein) and *Rps18* (ribosomal protein S18) were selected for their stable expression in mouse ovarian tissue across different developmental stages and treatment conditions, as validated in our previous work and other literature [69,70]. All primer sequences are listed in Table 1.

### 4.7. Telomerase Activity (TRAP) and Telomere Length Analysis

Telomerase activity in MII oocytes was assessed using the Telomeric Repeat Amplification Protocol (TRAP) via qPCR, as previously described [71]. For telomere length analysis, genomic DNA was extracted from pools of 50–100 MII oocytes using the QIAamp UCP DNA Micro Kit (56204, Qiagen, Hilden, Germany). Relative telomere length was determined via qPCR, comparing the amplification of telomeric repeats (Tel) to that of a reference gene (*Rn18s*), a multi-copy gene suitable for low-input samples [72]. Primer sequences are listed in Table 1.

### 4.8. Western Blotting

Ovarian protein lysates were prepared using RIPA buffer, and protein concentration was determined with a BCA Protein Assay Kit (23225, Thermo Fisher Scientific, Waltham, MA, USA). Proteins (20–30 µg) were denatured, separated by SDS-PAGE (10% or 12% gels), and transferred to a PVDF membrane. For the analysis of Cyp19a1 and Gdf9, 6 biological replicates from control mice and 7 biological replicates from fluoxetine-treated mice were initially analyzed. Similarly, for Sert, 6 biological replicates from control mice and 7 biological replicates from fluoxetine-treated mice were analyzed. Each biological sample was run in a single technical replicate. Membranes were blocked with 5% BSA and incubated with primary antibodies (Table 2). Following incubation with HRP-conjugated secondary antibodies (Table 2), chemiluminescence was detected using a Fusion-FX7 system (Vilber Lourmat, France). Band intensities were quantified using Image Lab software (Version 6.0.1, Bio-Rad Laboratories, Inc., Hercules, CA, USA) and normalized to β-actin or Hsp90 as loading controls. The final number of biological replicates included in the quantitative analysis for each protein is indicated in the respective figure legends (Figure 5).

### 4.9. Statistical Analysis

Statistical analysis was performed using GraphPad Prism 9.0 (GraphPad Software, San Diego, CA, USA). Data were first tested for normality using the Shapiro–Wilk test. For comparison of two groups, an unpaired Student’s t-test or Mann–Whitney U test was used for normally or non-normally distributed data, respectively. For multiple group comparisons, one-way or two-way ANOVA followed by an appropriate post hoc test (e.g., Tukey’s or Sidak’s) was applied. Data are presented as the mean ± SEM. A *p*-value of < 0.05 was considered statistically significant.

## 5. Conclusions

Our research demonstrates that fluoxetine has a potential impact on ovarian function, and we propose a potential mechanism centered on the oocyte. We suggest a proposed mechanism: fluoxetine inhibits the oocyte’s serotonin transporter (SERT), leading to a significant downregulation of the pivotal oocyte-derived factor GDF9. This disruption, in turn, dysregulates gene expression in surrounding somatic cells, ultimately manifesting as compromised oocyte competence and reduced ovulatory potential. Critically, the heightened vulnerability observed in prepubertal females indicates that early follicular development is a window of particular susceptibility to SSRI-induced damage. These findings carry significant clinical weight, raising concerns about the potential impact of SSRI therapy on the establishment and preservation of the ovarian reserve in adolescents and underscoring the need for informed counseling when prescribing these medications to women of reproductive age.

## Figures and Tables

**Table 1:** Primer sequences used for qPCR analysis.

Gene Name	NCBI Gene ID	Forward Primer	Tann, °C	Reverse Primer	Tann, °C
*Bmp6*	12161	ACCGTACTTTGTGGCAGAGC	68.3	GAAAAGGCAAAGAGCAGAGTTAG	66.4
*Bmp15*	12155	GAATCTGATGCCTCTTGTCCTT	69.5	ATGGCATGGTTGGGTGAAT	63.2
*Cyp11a1*	13070	GCCTGGAGCCATCAAGAACT	70.3	GAAAAGCGGAATAGGTCATCACT	66.4
*Cyp17a1*	13074	CGGTGGCCCCCTTGCTCA	81.6	GGCTGGTCCCATTCATTTTTATCGTG	69.7
*Cyp19a1*	13075	TCTCCTCATCAAACCAAACATCTTCT	69.1	CAGTTGCAAAATCCATACAGTCTTCC	69.7
*Fshb*	14308	TCGCCCACCCTTGTCCT	74.4	CTGGCCCTGGCACTCCTA	66.8
*Fshr*	14309	TGCTACACCCACATCTACCTCACA	70.3	GGATCTTGGCCTTGGACACAGT	69.5
*Gdf9*	14566	GCCTCCCCGACCTTTAGA	71.5	TGCCTCAGACTCCACATTTTC	65.0
*Has2*	15117	GCGGAAGAAGGGACAACA	69.3	TGCGGTGCCACAATACTG	64.5
*Igf1*	16000	GACCGAGGGGCTTTTACTTCAACA	73.9	GGCGCTGGGCACGGATAG	71.3
*Lhb*	16866	TGGCCGCAGAGAATGAGTT	68.9	TGAGGGCTACAGGAAAGGAGAC	73.2
*Lhgr*	16867	CTCTCACCTATCTCCCTGTCAAAGTAA	69.4	TGTAAAAGCACCGGGTTCAATGT	68.2
*Ptgs2*	19225	CCCTCCGGTGTTTGTCCTT	67.5	CCTGCAGCATTTTTCATCTTGTA	64.6
*Rps18*	20084	AAGAAAATTCGAGCCCATAGAGG	67.8	TAACAGCAAAGGCCCAGAGACT	69.5
*Rn18s*	19791	GACTCAACACGGGAAACCTCA	71.8	CAAATCGCTCCACCAACTAAGA	67.6
*Sert (Slc6a4)*	15567	GGGAGACCTGGGGCAAGAAG	74.8	CAGGGCGAGCTCCATGTAGAAGA	71.8
*Star*	20845	GCCCACTTTTCTGTCCCTTAT	68.9	CTGCCCTCGCTCACCTTA	64.5
*Tbp*	21374	GTAGCGGTGGCGGGTATCT	71.3	CGTCTTCAATGTTCTGGGTTATCT	67.0
*Tel*	-	CGGTTTGTTTGGGTTTGGGTTTGGGTTTGGGTTTGGGTT	76.2	GGCTTGCCTTACCCTTACCCTT ACCCTTACCCTTACCCT	80.0

**Table 2:** List of antibodies used for membrane staining in Western blotting.

Antibody	Manufacturer	Catalogue Number	Dilution
**Primary antibodies**			
Anti-β-actin, mouse	Sigma-Aldrich, St. Louis, MO, USA	A5441	1:10,000
Anti-Cyp19a1, rabbit	Abcam, Cambridge, Cambridgeshire, UK	ab18995	1:1000
Anti-Gdf9, goat	Thermo Fisher Scientific, Waltham, MA, USA	PA5-47924	1:200
Anti-Hsp90, rabbit	Sigma-Aldrich, St. Louis, MO, USA	SAB4300541	1:5000
Anti-Sert, goat	Abcam, Cambridge, Cambridgeshire, UK	ab130130	1:5000
**Secondary antibodies**			
Horseradish peroxidase, IgG donkey, against goat	Sigma-Aldrich, St. Louis, MO, USA	SAB3700284	1:50,000
Horseradish peroxidase, IgG goat, against mouse	Jackson ImmunoResearch Labs, West Grove, PA, USA	115-035-003	1:50,000
Horseradish peroxidase, IgG goat, against rabbit	Jackson ImmunoResearch Labs, West Grove, PA, USA	111-035-003	1:50,000

## Data Availability

The original contributions presented in this study are included in the article. Further inquiries can be directed to the corresponding author.

## Abbreviations

The following abbreviations are used in this manuscript:

5-HT	5-hydroxytryptamine, serotonin
dpp	days postpartum
E2	17β-estradiol
FLX	fluoxetine
FSH	follicle-stimulating hormone
GV	germinal vesicle
hCG	human chorionic gonadotropin
LH	luteinizing hormone
MII	metaphase II
NSN	non-surrounded nucleolus-like bodies
PMSG	pregnant mare serum gonadotropin
RPL	relative protein levels
RQ	relative gene expression
SN	surrounded nucleolus-like bodies
SSRI	selective serotonin reuptake inhibitor
VH	vehicle
